# A Faster and Lighter Detection Method for Foreign Objects in Coal Mine Belt Conveyors

**DOI:** 10.3390/s23146276

**Published:** 2023-07-10

**Authors:** Bingxin Luo, Ziming Kou, Cong Han, Juan Wu, Shaowei Liu

**Affiliations:** 1 School of Mechanical Engineering, Taiyuan University of Technology, Taiyuan 030024, China; tyutedu0709@163.com (B.L.); hancong@tyut.edu.cn (C.H.); wujuanz@163.com (J.W.); 15530080650@163.com (S.L.); 2 Shanxi Provincial Engineering Laboratory for Mine Fluid Control, Taiyuan 030024, China

**Keywords:** belt conveyor, foreign object recognition, parameterless attention mechanism, deep learning, lightweight network

## Abstract

Coal flow in belt conveyors is often mixed with foreign objects, such as anchor rods, angle irons, wooden bars, gangue, and large coal chunks, leading to belt tearing, blockages at transfer points, or even belt breakage. Fast and effective detection of these foreign objects is vital to ensure belt conveyors’ safe and smooth operation. This paper proposes an improved YOLOv5-based method for rapid and low-parameter detection and recognition of non-coal foreign objects. Firstly, a new dataset containing foreign objects on conveyor belts is established for training and testing. Considering the high-speed operation of belt conveyors and the increased demands for inspection robot data collection frequency and real-time algorithm processing, this study employs a dark channel dehazing method to preprocess the raw data collected by the inspection robot in harsh mining environments, thus enhancing image clarity. Subsequently, improvements are made to the backbone and neck of YOLOv5 to achieve a deep lightweight object detection network that ensures detection speed and accuracy. The experimental results demonstrate that the improved model achieves a detection accuracy of 94.9% on the proposed foreign object dataset. Compared to YOLOv5s, the model parameters, inference time, and computational load are reduced by 43.1%, 54.1%, and 43.6%, respectively, while the detection accuracy is improved by 2.5%. These findings are significant for enhancing the detection speed of foreign object recognition and facilitating its application in edge computing devices, thus ensuring belt conveyors’ safe and efficient operation.

## 1. Introduction

The underground mining environment in coal mines is complex [1], with coal flow in belt conveyors easily contaminated by foreign objects, such as anchor rods [2], angle irons [3], pallets [4], and gangue [5]. If these foreign objects are not promptly sorted out, they can lead to accidents, such as belt tearing, blockages at transfer points, or even belt breakage, severely impacting the safety and efficiency of coal mining operations [6]. Therefore, the rapid and accurate detection of non-coal foreign objects on the conveyor belt and their timely sorting can greatly prevent belt damage [7], prolong the service life of the conveyor belt, and further reduce the occurrence rate of coal mining accidents [8].

Traditional methods for foreign object detection include manual inspection [9], X-ray and dual-energy gamma-ray detection [10], and optical image-based detection [11]. However, manual inspection is often time-consuming and inefficient. X-ray and gamma-ray detection can achieve higher accuracy, but prolonged exposure of operators to radiation poses potential health risks [12]. On the other hand, optical image-based detection faces challenges in extracting features from complex backgrounds, and overly simplistic model construction leads to low detection accuracy [13].

In recent years, with the rapid development of computer devices and computer vision technologies, significant advancements have been made in deep learning theory, making it the mainstream research algorithm in object detection. Many researchers are dedicated to utilizing machine vision and deep learning methods for foreign object detection on conveyor belts.

Li Dongjun et al. [14] proposed a coal gangue layer detection framework based on a deep learning model and designed a Coal Gangue Region Proposal Network (CG-RPN) to extract features. The framework achieved a maximum detection accuracy of 98.3% on a custom dataset, which is 0.8% higher than the existing methods. This approach addressed the issue of low detection accuracy in traditional recognition methods. However, using the R-CNN backbone network made it challenging to rapidly identify foreign object features in the complex background of coal mines.

Xiao Dong et al. [15] proposed an object recognition method for foreign objects based on channel pruning of YOLOv3. They classified 346 collected samples of foreign objects, such as steel bars, plastic pipes, and wood, achieving a maximum detection accuracy of 92.6%. The model optimized the parameter and computational complexity of the original model through pruning strategies. However, the limited number of samples resulted in the need for further improvement in the training effectiveness of the model.

Yuanbin Wang et al. [16] proposed an improved foreign object recognition method based on Single Shot MultiBox Detector (SSD) and modified the model’s loss function. Compared to YOLOv3, their approach achieved a higher detection accuracy of 90.2%. However, the SSD-based detection method requires manual parameter setting, and it inadequately captures the features of small and fine-grained foreign objects at the edges.

Mengchao Zhang et al. [17] developed a deep lightweight model based on YOLOv4 using the Mobilenet lightweight network architecture. This approach improved the detection speed of non-coal foreign objects. However, the custom dataset used in their study exhibited an imbalanced sample distribution, with a severe label imbalance between the coal gangue and other foreign objects, resulting in limited generalization performance.

Qinghua Mao et al. [18] integrated adaptive spatial features and the Convolutional Block Attention Module (CBAM) attention mechanism into the YOLOv5 network model. This integration allows for the complete extraction of multi-level feature information from foreign object images, accurately identifying foreign objects. In their proposed foreign object dataset, they achieved a maximum detection accuracy of 95.09%. However, introducing the CBAM attention mechanism requires additional computational resources, resulting in higher computational complexity and affecting the speed of foreign object recognition and detection.

Zhiling Ren et al. [19] proposed an object recognition algorithm based on CenterNet to address the issue of large variations and uneven distribution in foreign object types. This algorithm reduces the model’s false positive rate. However, CenterNet requires setting different parameters for different datasets, leading to poor generalization performance. Additionally, it increases computational complexity and generates excessive data redundancy.

Qinghua Mao et al. [20] proposed an improved method for foreign object recognition in coal mine belt conveyor systems based on YOLOv7. This method enhances the recognition speed by replacing regular convolutions in the backbone feature extraction network with depthwise separable convolutions. The detection speed achieved was 25.64 frames per second. However, the extensive use of depthwise separable convolutions led to decreased recognition accuracy, with a precision rate of only 92.8%.

With the rapid development of high-speed belt conveyors, there is a need for faster frame rates to capture clear images. On the one hand, a higher frame rate allows underground cameras to detect conveyor belt foreign objects more frequently within the same detection range, thereby improving detection accuracy and reducing the rate of missed detections. On the other hand, clearer input images enable faster processing by the network model, providing edge computing devices with more time. Hence, this imposes higher requirements on the real-time performance of the algorithm. Furthermore, edge devices deployed locally at the coal mine face limitations in computational capabilities. As a result, when simultaneously processing multiple video streams, there is a preference for “hardware-friendly algorithms”, which have fewer model parameters and lower network computational requirements. In coal mining, erroneous or delayed judgments can lead to incorrect decisions, posing certain safety risks or economic losses. In order to improve the real-time detection of foreign matter, effectively reduce the dependence of complex networks on hardware performance, and provide a convenient new method for the safe operation of belt conveyors, from the above perspective, this article proposes a faster and fewer-parameter non-coal foreign object recognition and detection method for belt conveyors based on improved YOLOv5.

The proposed method takes into full consideration the impact of dust, noise, and fog in underground coal mines on the model’s detection accuracy. A deep lightweight object detection network is achieved by improving the backbone and neck of YOLOv5, ensuring detection speed and accuracy while reducing computational model complexity. This effectively enhances the generalization ability of the algorithm and increases its likelihood of application in the field.

The rest of this paper is organized as follows: Section 2 describes the data preparation process, Section 3 presents the algorithm improvements, Section 4 analyzes the experimental results and relevant discussions, and Section 5 concludes the study with a summary of the findings and considers future work.

## 2. Data Preparation

**Figure 1:**
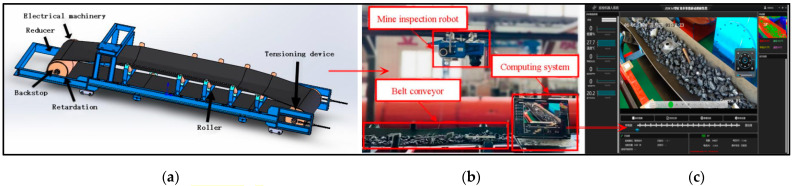
Experimental environment and hardware facilities. (**a**) 3-D model of belt conveyor belt; (**b**) experimental equipment diagram; (**c**) upper computer system of mining inspection robot.

Data, computing power, and algorithms are the three fundamental elements of artificial intelligence [21,22]. In the context of fixed computing power and algorithms, the quantity and quality of data directly determine the final detection performance. Due to the specific nature of the detection target, no existing public dataset has been found that specifically addresses the detection of foreign objects on conveyor belts. Therefore, the dataset used in the experiments was obtained from video images captured by the inspection robot (model ZDX12) of Boshitong Company in Taiyuan, Shanxi Province, China, during the operation of the conveyor belt. The conveyor belt had a running speed of 4m/s, and the inspection robot captured frames at 40 frames/s. The image resolution was set to 1920 × 1080. The experimental environment and hardware facilities are shown in Figure 1 and Table 1. However, at this resolution and testing scenario, a significant amount of memory and bandwidth are required for transmission. Implementing this in an ©ndustrial setting, especially edge computing, requires support from high hardware computing capabilities. In order to reduce the computational cost and improve the network’s performance, this paper resized the image resolution to 640 × 640 using Python batch processing. The images were labeled using Labelme software(version number: 5.1.1), and the dataset was stored in the VOC2007 format.

To enhance the recognition capability of the improved YOLOv5 network model for detecting objects under different angles and lighting conditions, data augmentation techniques, such as horizontal flipping, mirror flipping, and brightness adjustment, were applied to all images in the dataset. After data augmentation, the dataset contains 17,483 foreign object image samples and 44,480 data labels.

**Figure 2:**
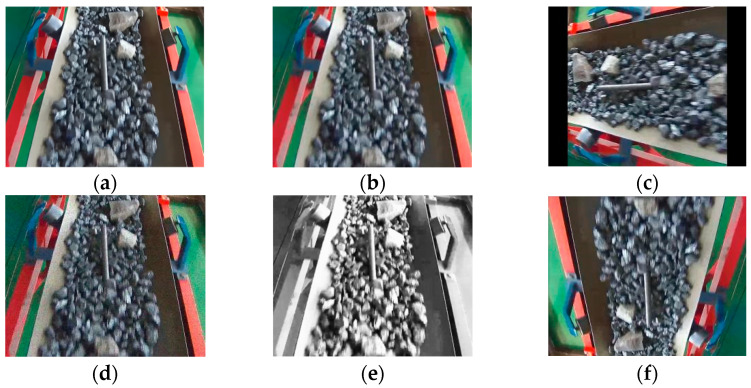
Partial image enhancement effect. (**a**) Original image; (**b**) random flip; (**c**) random cropping; (**d**) noise enhancement; (**e**) grayscale enhancement; (**f**) random rotation.

**Figure 3:**
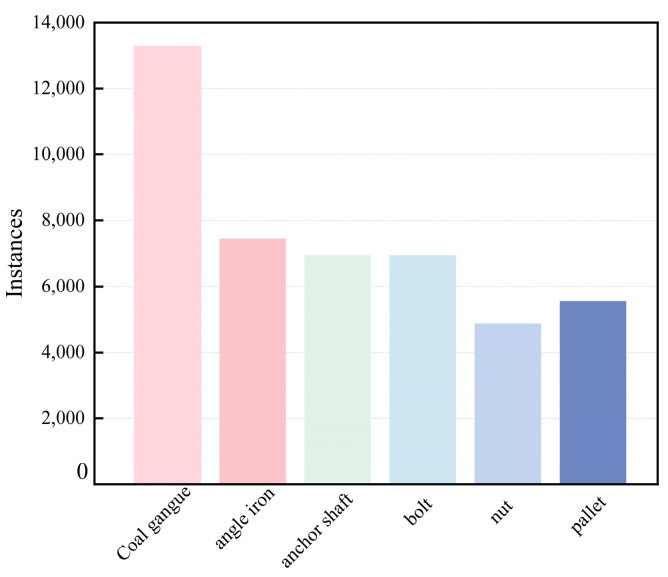
Distribution of types of foreign object labels.

During the training process, all image samples were divided into a training set (12,238 images), validation set (3496 images), and test set (1749 images) in a ratio of 7:2:1. The dataset consists of various objects, including anchor rods, angle irons, pallets, gangue rocks, nuts, and screws. Some examples of augmented images are shown in Figure 2, and the classification of non-coal objects is illustrated in Figure 3.

Based on the statistical analysis combined with Figure 3, among the 44,480 data labels, a significant proportion is occupied by coal gangue due to its generation throughout various stages of coal mining and coal preparation processes. On the other hand, the distribution of other foreign objects, such as anchor rods, angle irons, nuts, trays, and screws, is relatively uniform.

## 3. Improvement of Algorithm

### 3.1. Video Image Preprocessing Algorithm

Considering the uneven lighting, noise, and foggy conditions in underground coal mines, data collection and foreign object detection pose significant challenges. To ensure accurate image annotation and precise identification of foreign objects, this study conducted preprocessing on the augmented dataset of 17,483 images. This preprocessing step aimed to mitigate the impact of adverse environmental factors on detection accuracy.

The dark channel dehazing algorithm [23] is based on observing haze-free images. For any image J, its dark channel is expressed as [24]:

(1) $$J^{dark}\left(x\right)=\underset{y\in \Omega \left(x\right)}{\min}\left(\underset{c\in \left(r,g,b\right)}{\min}J^{c}\left(y\right)\right)$$

where *J^dark^*(*x*) represents the dark channel map, *C* represents one of the channels *R*, *G*, and *B*, *x* represents a pixel point in the map, *Ω*(*x*) represents the small area around the pixel point.

The specific steps of the algorithm are as follows: first, obtain the dark channel map, and then find the value of the highest brightness point at the corresponding position in the original fog map *I*(*x*) as the atmospheric light value A. Then, set the lower limit value *t*_0_ (generally 0.1) for the transmittance. When *t* is less than *t*_0_, take *t* = *t*_0_ to avoid the excessive white field of the restored original fog image. Finally, the transmittance and atmospheric light values are substituted into the formula to obtain the restored image. The recovery formula is as follows (2):

(2) $$J\left(x\right)=\frac{I\left(x\right)}{\max\left(t\left(x\right),t_{0}\right)}+A$$

where *I*(*x*) represents the foggy image, *J*(*x*) represents the surface fog-free image, *A* represents the global atmospheric light value, *t*(*x*) represents transmittance.

In this way, images with defogging can be output by combining the results of dark channel calculation with the atmospheric scattering model.

### 3.2. Improved Foreign Object Recognition Method of YOLOv5 Algorithm

The YOLOv5s algorithm effectively extracts image features and achieves object detection. However, the production process in coal mines involves complex environmental conditions and has higher demands for the speed and accuracy of non-coal foreign object detection and hardware deployment. To address this, this study proposes a lightweight feature aggregation approach that optimizes parameter quantity and computational complexity while ensuring rich feature representation. 

**Figure 4:**
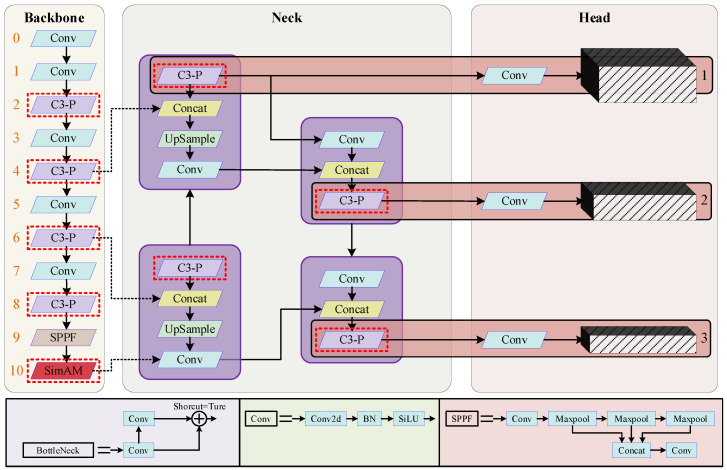
Improved YOLOv5 network model framework.

Specifically, to enhance the model’s ability to handle complex backgrounds and extract features of foreign objects, a lightweight attention mechanism called Simple Attention Module (SimAM) [25] is introduced to the feature extraction network. Furthermore, a novel lightweight convolution (PConv) [26] is proposed, and the C3 module of the network is redesigned to optimize the model’s parameter quantity and computational speed. Additionally, the bounding box regression loss function CIoU is replaced with SIoU, leading to improved convergence speed and detection accuracy of the model. The modified architecture, as shown in Figure 4, highlights the improved components with red dashed boxes. Further details about the improvements are elaborated in subsequent subsections.

#### 3.2.1. SimAM Attention Mechanism

Human attention is one of the most crucial selection mechanisms, prioritizing the processing of task-relevant information while attenuating irrelevant signals. Inspired by the attention mechanisms in human visual processing, researchers have designed similar attention modules in convolutional neural networks [27,28]. Existing attention modules are typically integrated into each block to improve the output of the preceding layer. This refinement process often occurs along the channel or spatial dimensions, generating 1-D or 2-D weights and treating neurons at each channel or spatial position equally. However, this approach may restrict their ability to learn more discriminative cues, for example, attention mechanisms such as SE [29], CBAM [30], CA [31], etc. The SE attention mechanism only considers attention along the channel dimension and cannot capture spatial attention. It is suitable for scenarios with many channels but may not perform as well as other attention mechanisms in cases with fewer channels. The CBAM attention mechanism requires additional computations, resulting in significant computational overhead for smaller feature maps. The CA attention mechanism also incurs additional computations and has a high computational cost. Furthermore, since it requires computing attention weights over the entire feature map, it cannot capture long-range dependencies.

In YOLOv5s, pyramid feature extraction is adopted as the backbone network [32]. However, in the operation of underground conveyor belts in coal mines, there are various non-coal foreign matter features that can interfere with the recognition process. During the recognition process of the network model, numerous noise signals are present, which can be propagated during the model’s learning process. As the number of network layers increases, the weights of the noise signals in the feature maps also increase, ultimately resulting in negative impacts on the model.

**Figure 5:**
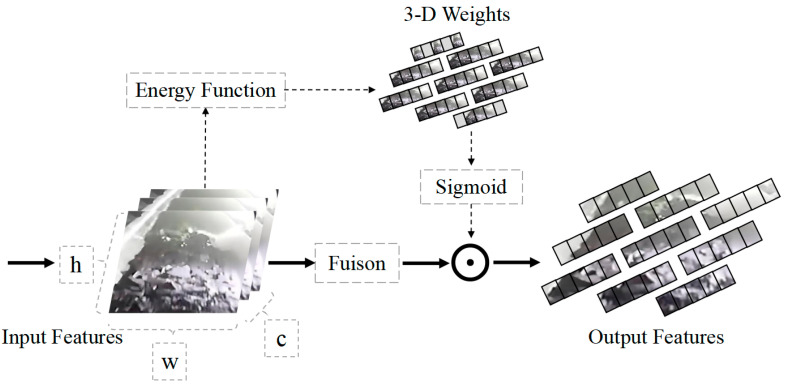
Schematic diagram of SimAM weight assignment.

Therefore, in this study, the SimAM attention mechanism is added after the SPPF layer of the backbone network to enhance its feature extraction capability and improve the representation power of the features. Compared to channel attention mechanisms and spatial attention mechanisms, SimAM assigns higher weights to neurons that contain more crucial information for visual-related tasks without the need for additional sub-network structures. By generating spatial inhibition among neighboring neurons of the objects, the interference from complex backgrounds in underground coal mines on object recognition is reduced, and the crucial features of the objects are highlighted, thereby enhancing the ability to extract key features of the objects. SimAM achieves this without introducing additional parameters while considering the spatial and channel dimensions’ correlations. The allocation principle of attention weights in SimAM is illustrated in Figure 5.

The 3-D weight resolution process is as follows: first, the importance of a single neuron needs to be estimated, and we measure the importance of a neuron by the linear differentiability between the target neuron and other neurons. The minimum energy of each neuron is calculated by Equations (1)–(3) based on the knowledge of visual neuroscience [33].

(3) $$e_{t}^{*}=\frac{4\left({\hat{\sigma }}^{2}+\lambda \right)}{\left(t-{\hat{\mu }}^{2}\right)+2{\hat{\sigma }}^{2}+2\lambda }$$

(4) $$\hat{\mu }=\frac{1}{M}\sum_{i=1}^{M}x_{i}$$

(5) $${\hat{\sigma }}^{2}=\frac{1}{M}\sum_{i=1}^{M}{\left(x_{i}-\hat{\mu }\right)}^{2}$$

where *λ* is the hyperparameter, *t* is the neuron of the target foreign body, $\hat{\mu }$ and ${\hat{\sigma }}^{2}$ are the mean and variance of all neurons in the channel, *x_i_* is the other neurons in the *i*-th channel that input the feature map.

Therefore, the importance of each neuron can be obtained by 1/$e_{t}^{*}$. Then, based on the attention modulation observed in the mammalian brain, which often manifests as a gain effect on neuronal responses, a scaling operator is used instead of an addition to refine the features, ensuring that each neuron is assigned a unique weight [34]. The calculation formula is as follows:

(6) $$Y=sigmoid\left(\frac{1}{E\left(x\right)}\right)⊙X$$

where *E* is the energy function across channels and spatial dimensions. The lower the energy, the higher the discrimination between the target foreign object neuron and the adjacent neuron. In order to prevent the *E* value from being too large, the Sigmoid function is used to suppress attention. *Y* represents the enhanced feature map of the foreign object, ⊙ represents the dot product operation, and *X* represents the input foreign object feature map.

#### 3.2.2. Lightweight Convolution Module C3-PConv

Floating-point operations (FLOPs) represent the number of floating-point calculations and can be used to measure the complexity of algorithms and models. A larger number of FLOPs indicates higher computational complexity and model complexity. In conventional convolution (Conv), all three channels are simultaneously processed, and the number of convolutional filters equals the number of output channels, while the number of channels in the filters equals the number of input channels. In YOLOv5s, the excessive use of the C3 module, which consists of multiple densely connected standard convolutions, and the excessive use of Conv for feature extraction may lead to an accumulation of parameters and redundant features. As the depth of the network increases, the effect becomes more significant, which further affects the speed of foreign object detection in coal mines.

**Figure 6:**
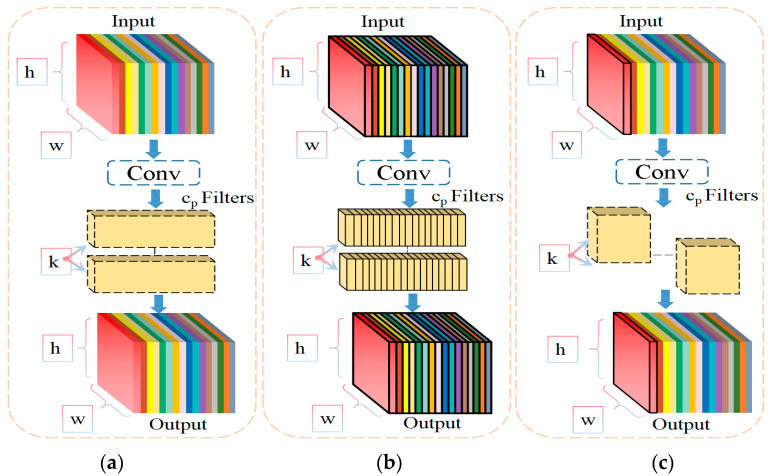
Comparison of PConv with other convolutional structures. (**a**) Conv; (**b**) DWConv; (**c**) PConv.

DWConv [35] is a widely used lightweight convolution method that reduces the number of parameters and FLOPs through filter redundancy. For input *I* ∈ *R^c×h×w^*, DWConv uses *c* filters *W* ∈ *R^k^^×k^* to calculate output *O* ∈ *R^c×h×w^*. Figure 6b shows that each filter slides spatially on one input channel and contributes one output channel. Compared to a normal convolutional Conv with *h × w × k*^2^ *× c*^2^, its FLOPs are as low as *h × w × k*^2^ *× c*:

(7) $$\frac{FLOPs_{DWConv}}{FLOPs_{Conv}}=\frac{h\times w\times k^{2}\times c^{}}{h\times w\times k^{2}\times c^{2}}=\frac{1}{c}$$

Although DWConv can effectively reduce FLOPs, it cannot simply replace the normal convolutional Conv, which causes severe accuracy degradation. Therefore, in practice, when the network width *c′* of DWConv is increased to (*c* < *c′*) to compensate for the accuracy degradation, it increases the memory requirements of the computation and thus slows down the overall computation [36]. For the deployed hardware devices, the number of memory accesses is escalated to

(8) $$h\times w\times 2c^{\prime }+k^{2}\times c^{2}\approx h\times w\times 2c^{\prime }$$

higher than the regular Conv, i.e.,

(9) $$h\times w\times 2c+k^{2}\times c^{2}\approx h\times w\times 2c$$

**Figure 7:**
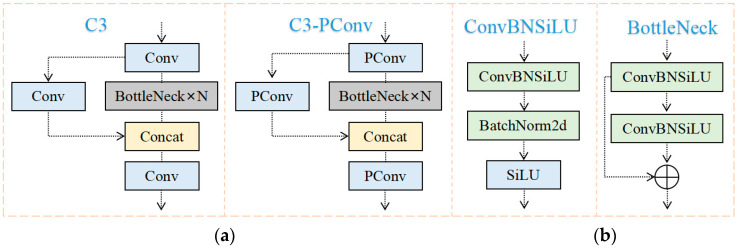
C3-PConv structure and C3 structure. (**a**) C3; (**b**) C3-PConv.

This paper introduces PConv instead of standard convolution and constructs a C3-PConv module, whose structure is shown in Figure 7, which can improve the model detection speed while ensuring detection accuracy. As depicted in Figure 6c, PConv employs regular convolution for spatial feature extraction on selected input channels while keeping the remaining channels unaltered. For continuous or regular memory access, calculate the first or last continuous c_p_ channel to represent the entire feature map. Without loss of generality, it is considered that the input and output characteristic maps have the same number of channels. At this time, the FLOPs of PConv are only

*h* × *w* × *k*^2^ × *c_p_*^2^ (10)

with a typical partial ratio, $r=\frac{c_{p}}{c}=\frac{1}{4}$; the FLOPs of a PConv are only $\frac{1}{16}$ of a regular Conv. In addition, PConv has a smaller access volume, i.e.,

(11) $$h\times w\times 2c_{p}+k^{2}\times c_{p}^{2}\approx h\times w\times 2c_{p},$$

which is only 1/4 of a regular Conv for r = 1/4. 

C3-PConv has the potential advantage of reducing computational redundancy and memory access compared to C3 modules. This is also verified in the fourth part of the experiments.

#### 3.2.3. SIoU Loss Function

The loss function serves to quantify the disparity between the model and actual data. In the original YOLOv5s network, the border regression loss utilizes the Complete Intersection over Union (CIOU) metric [37], which is computed as follows:

(12) $$CIoU=1-IOU+\frac{\rho^{2}\left(p,p^{gt}\right)}{\left|c^{2}\right|}+\frac{V^{2}}{\left(1-IoU\right)+V}$$

(13) $$V=\frac{4}{\pi }{\left(\arctan\frac{w^{gt}}{h^{gt}}-\arctan\frac{w}{h}\right)}^{2}$$

where *p* and *p^gt^* make the centroids of the prediction frame *B* and the true frame *B^gt^*, *c* is the diagonal length, *ρ* denotes the Euclidean distance, and *V* denotes the consistency of the metric aspect ratio.

Although the CIoU loss function considers the overlap area, center point distance, and aspect ratio during bounding box regression, it does not directly account for the actual differences in width and height between the predicted and ground truth boxes. This limitation sometimes hampers model optimization, resulting in slow convergence and imprecise predicted boxes. To enhance both the speed and accuracy of non-coal foreign object detection, this study employs SIoU as the boundary frame regression loss calculation using the following equation [38]:

(14) $$\Delta =2-e^{-\gamma \frac{xgt-x}{cw}}-e^{-\gamma \frac{ygt-y}{ch}}$$

(15) $$\Omega ={\left(1-e^{-\omega w}\right)}^{2}+{\left(1-e^{-\omega h}\right)}^{2}$$

(16) $$SIoU=1-IoU+\frac{\Omega +\Delta }{2}$$

where $\omega_{W}=\frac{\left|w-w^{gt}\right|}{\max\left(w,w^{gt}\right)}$, $\omega_{h}=\frac{\left|h-h^{gt}\right|}{\max\left(h,h^{gt}\right)}$, $\gamma =1+2\sin^{2}\left(\arcsin\left(\frac{\max\left(y^{gt},y\right)-\max\left(x^{gt},x\right)}{\sqrt{{\left(x^{gt}-x\right)}^{2}+{\left(y^{gt}-y\right)}^{2}}}\right)-\frac{\pi }{4}\right)$.

**Figure 8:**
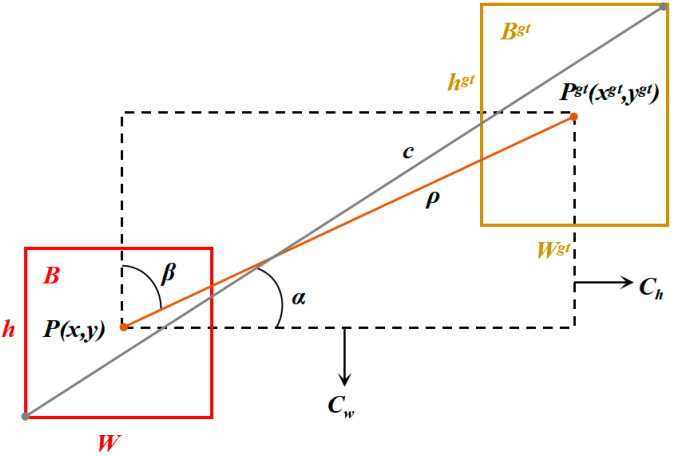
Real box and predicted box.

On the basis of considering the distance, overlapping area, and aspect ratio between the frame centers, SIoU adds angle loss, as shown in Figure 8, to effectively reduce the total degree of freedom lost so that the prediction box can quickly move to the nearest axis, accelerating training convergence and frame regression effect.

## 4. Analysis of Experimental Results

### 4.1. Environmental Configuration

In order to ensure the scientific reliability of the experimental conclusions, all the experiments were conducted using the Stochastic Gradient Descent (SGD) optimizer for parameter updates. The experiments were iterated for 300 epochs with a batch size of 16. The input image size was set to 640 × 640. The initial learning rate was set to 0.01, and a weight decay coefficient of 0.0005 was applied to prevent overfitting during the training process. A momentum coefficient of 0.937 was used to prevent the model from becoming trapped in local optima or skipping over the global optimum. The environment configuration is shown in Table 2.

### 4.2. Analysis of Image Preprocessing Results

**Figure 9:**
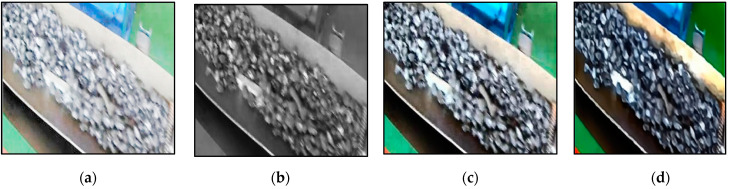
Comparison of the effect before and after pre-processing. (**a**) Original image; (**b**) grayscale enhancement; (**c**) histogram equalization; (**d**) dark channel defogging.

This paper compares several image enhancement algorithms commonly applied in coal mining, including grayscale enhancement algorithms, histogram equalization algorithms, and dark-channel-based image-defogging algorithms. The experimental results demonstrate that the dark-channel-based defogging algorithm provides the best pre-processing of the acquired data, producing sharper contours and better highlighting the features of foreign objects and backgrounds. The effect of partial enhancement is shown in Figure 9. From a subjective perspective, the original image is relatively blurry regarding the contour of foreign objects. The enhanced dark channel defogging image reduces noise and sharpening, which is more conducive to the network model’s extracting non-coal foreign object features.

To objectively evaluate the effectiveness of the enhanced images, we utilized peak signal-to-noise ratio (PSNR) and structural similarity (SSIM) with information entropy as objective evaluation criteria [39]. These objective evaluation indicators are widely used to measure the effectiveness of image processing in coal mines [40,41,42]. The formulas are as follows:

(17) $$MSE=\frac{1}{H\times W}\sum_{i=1}^{H}\sum_{j=1}^{W}{\left(X\left(i,j\right)-Y\left(i,j\right)\right)}^{2}$$

(18) $$PSNR=10\log_{10}\left(\frac{{\left(2^{n}-1\right)}^{2}}{MSE}\right)$$

where *MSE* represents the mean square error of the current image *X* and the reference image *Y, H* and *W* represent the height and width of the image, *N* is the number of bits per pixel. *PSNR* is expressed in *dB*, with higher values indicating less image distortion.

(19) $$SSIM\left(x,y\right)=\frac{\left(2\mu_{x}\mu_{y}+c_{1}\right)\left(2\sigma_{xy}+c_{2}\right)}{\left(\mu_{x}^{2}+\mu_{y}^{2}+c_{1}\right)\left(\sigma_{x}^{2}+\sigma_{y}^{2}+c_{2}\right)}$$

where *μ* and *σ* represent the mean and variance of image blocks, *σ_xy_* represents the covariance between image blocks *x* and image blocks *y, c*_1_ and *c*_2_ are constants. The value of *SSIM* is generally at (0, 1), and, the larger it is, the better the image enhancement effect.

(20) $$H=\sum_{i=0}^{255}P_{i}\log P_{i}$$

where *P_i_* represents the probability of a certain gray value appearing in the image, *H* represents the result of the information entropy calculation. The higher the entropy value, the brighter the image color and the clearer the contour.

**Figure 10:**
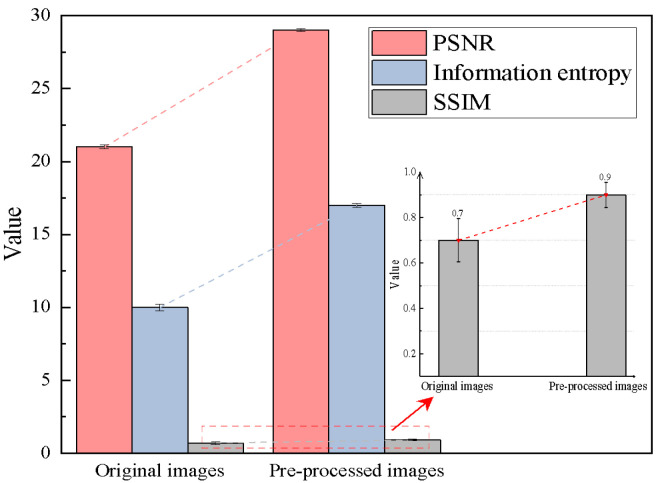
Image pre-processing quality assessment.

The average PSNR, SSIM, and information entropy values of the preprocessed image are shown in Figure 10 and Table 3. The results indicate that the preprocessed image is clearer than the original image, as evidenced by its higher PSNR, SSIM, and information entropy.

### 4.3. Analysis of Improved YOLOv5 Algorithm Results

In this study, YOLOv5s was selected as the baseline model, and the improved model was compared with the baseline. The significance of the algorithm improvement was evaluated based on metrics such as precision (P), recall (R), mean average precision (mAP), parameter amount, and inference speed. The formulas for precision, recall, and mAP are as follows:

(21) $$P=\frac{TP}{\left(TP+FP\right)}$$

(22) $$R=\frac{TP}{\left(TP+FN\right)}$$

(23) $$AP=\int_{0}^{1}P\left(R\right)dR$$

where *TP* represents the number of true samples, *FP* represents the number of false positive samples, *FN* represents the number of true negative samples.

#### 4.3.1. Analysis of C3-PConv Module Effectiveness Results

To validate the effectiveness of the C3-PConv module, referred to as C3-P for brevity, we seriatim replaced the C3 modules with the C3-P modules. The results are shown in Table 4, where the four C3 modules in the backbone are denoted as A1, A2, A3, and A4 in the order of feature map transmission. The C3 modules in the neck are denoted as B1, B2, B3, and B4. The first row in the table represents the results of the original YOLOv5s model (mAP: 92.4%; parameters: 7.2 M; inference time: 0.0089 s; FLOPs: 16.5G). With the increase in C3-P modules in the backbone layer, the recognition accuracy is slightly improved. Using the C3-P module (mAP: 92.6%; parameters: 7.0 M; inference time: 0.0061 s; FLOPs: 9.1 G) in the entire backbone network improves mAP by 0.2% compared with the baseline and saves 5.7% and 22.5% in parameters and inference time, and the model calculation amount (FLOPs) was reduced by 24.2%.

The neck layer serves as a key component for feature fusion. When replacing the C3-P module at position B1 in the neck layer, there is a slight increase in model computational complexity and inference time. This is because, when fusing shallow-level features, the sizes of coal and gangue objects relative to the entire image are relatively small, leading to redundancy in feature extraction for larger objects. As the number of C3-P modules increases, there is a gradual reduction in computational redundancy and memory access (mAP: 93.1%; parameters: 4.1 M; inference time: 0.0043 s; FLOPs: 9.1 G). Compared with the baseline, the accuracy of foreign object recognition has been improved by 0.7%, and the number of parameters, inference time, and model computation have been reduced by 44.6%, 51.7%, and 44.8%, respectively. In summary, the rationality of embedding the C3-P module in this article has been verified.

In addition, we compared the effectiveness of using deep separable convolution DWConv, and the results are shown in Table 5. DWConv was embedded in the C3 module, abbreviated as the C3-D module. From Table 5, it can be seen that, although the C3-D module has a significant effect on improving accuracy (mAP: 94.2%), the inference time and model computation amount have significantly increased (inference time: 0.0079 s; FLOPs: 15.5), which is contrary to our original intention to improve the network and also goes against the high real-time detection requirements of the coal mine underground deployment. In summary, the effectiveness of this paper for embedding C3-P modules is verified.

#### 4.3.2. Analysis of Foreign Object Detection Results

**Figure 11:**
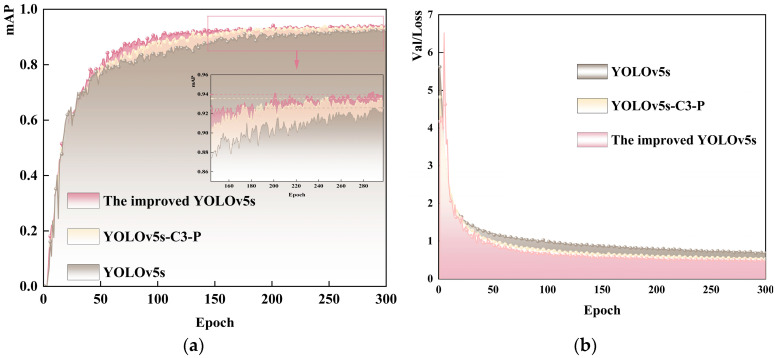
Model training results. (**a**) Improved model mAP curve comparison chart; (**b**) improved model loss curve comparison chart.

Figure 11a,b compare foreign object recognition accuracy and loss function convergence results before and after YOLOv5s improvement. The results indicate that the improved model converges faster and better than the baseline model. Specifically, the model converges significantly after approximately 50 iterations, achieving a detection precision of up to 94.9%, 2.5% higher than the baseline YOLOv5s.

Table 6, Table 7 and Table 8 display the P, R, and mAP values before and after the improvement of the model, respectively. The comparison shows that the improved model has slightly enhanced overall recognition of P, R, and mAP. The recognition precision for bolts, angle iron, anchor shafts, and nuts was improved by 4.4%, 1.9%, 0.3%, and 0.3%, respectively. The recall rates also showed an improvement of 12.1%, 0.1%, 1.5%, 8.4%, and 2.4% for bolts, angle iron, gangue, pallets, and nuts, respectively. Although there was a slight decrease in the detection accuracy of some foreign objects, such as a 0.2% and 0.1% decrease in the identification precision of gangue and pallets, and a 0.2% decrease in the recall rate of anchor shafts, the mAP values of various foreign objects increased by 7.7%, 1.7%, 0.9%, 0.8%, 0.8%, and 3.4%, respectively. Overall, the accuracy of the model detection after improvement showed promising results.

**Figure 12:**
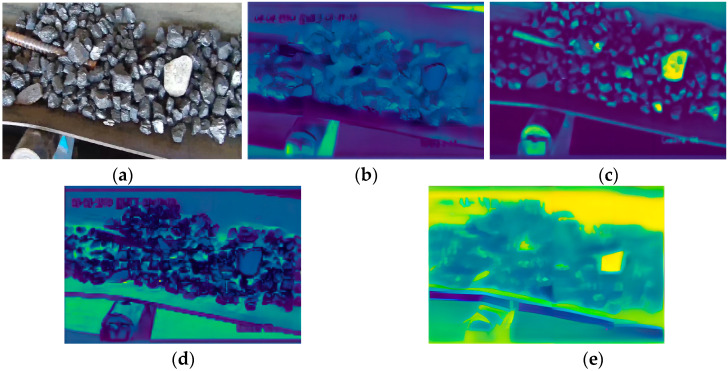
Activation diagrams of different feature layer classes. (**a**) Original image; (**b**,**c**) effect of shallow feature extraction before and after improvement; (**d**,**e**) effect of deep feature extraction before and after improvement.

The network model prefers to use the location of the features, and we hope that the improved model will be more effective in the feature extraction of foreign objects. In order to more intuitively analyze and compare the effectiveness of the improved model in identifying foreign objects before and after, feature extraction class activation maps are added in this paper. Figure 12a shows an original image from the data sample, (b) and (c) show the same shallow feature extraction effect before and after the improvement, and (d) and (e) show the same profound feature extraction effect of the network before and after the improvement.

By comparison, it can be found that the YOLOv5s network model extracts foreign object features vaguely in the shallow feature extraction and pays more attention to non-foreign object features, such as belts and rollers, resulting in missed detection during overall feature extraction. In contrast, the improved network model has clearer feature extraction for foreign objects, and deep feature extraction covers the foreign object detection area. 

**Figure 13:**
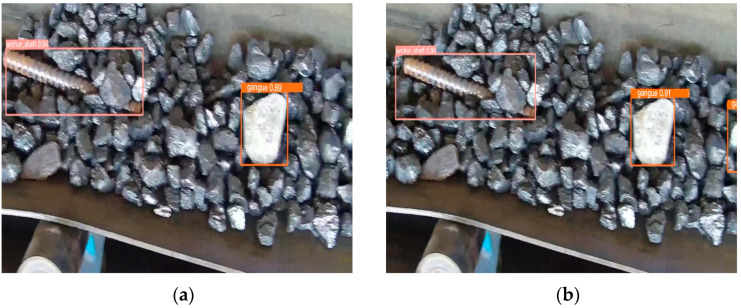
Partial recognition of foreign object detection results before and after improvement. (**a**) Recognition results of the model before improvement; (**b**) model recognition results after improvement.

Figure 13 shows the detection results of the corresponding feature class activation graphs, where (a) and (b) indicate the detection results of the improved before and after models, respectively. Comparing the detection result plots, the original network may have missed detecting small foreign objects at the edges and corners, and the improved model has a significant recognition effect.

#### 4.3.3. Analysis of Parameter Quantity and Inference Speed Results

Balancing recognition accuracy and speed is a challenging task. In order to verify the significance of the new model, it was first compared with the baseline YOLOv5s, with a slight increase of 2.5% in mAP, a reduction of 43.1% in parameter amount, and 56.1% in inference time, respectively. The improved model is referred to as YOLOv5-PS, and, on this basis, YOLOv5m, YOLOv5l, and YOLOv5x were compared, as shown in Table 9. Although YOLOv5x achieves high detection accuracy (mAP: 96.9%), the associated computational burden (FLOPs: 205.7 G) and parameter amount (parameters: 86.7 M) contradict the initial intention of fast detection deployment in coal mine applications. The YOLOv5-PS model achieves a parameter amount of 4.1 M, an inference speed of 0.0043 s per frame, and only 9.1 FLOPs in the proposed coal mine anomaly detection. Compared to other YOLOv5 models, such as 5m, 5l, and 5x, YOLOv5-PS exhibits significant advantages regarding parameter amount, inference time, and model complexity.

To further validate the reliability of the improved model, this study also compared it with several representative network models, including SSD [43], Centernet [44], ConvNeXt [45], Shufflenet [46], Mobilenetv3 [47], and C2f [48], as well as mainstream attention mechanisms, such as SE, CBAM, and CA.

**Figure 14:**
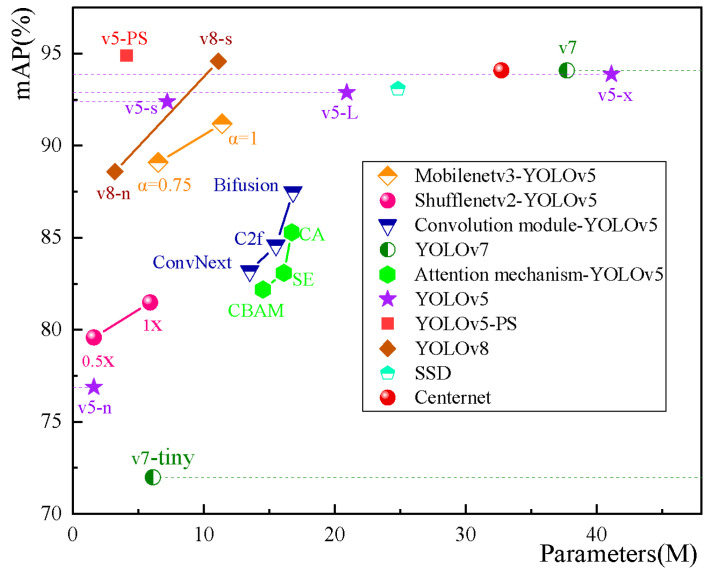
Comparison chart of mainstream models.

The comparative results are shown in Figure 14. Although the Mobilenetv3 and Shufflenev2 network models reduce the number of parameters, they also suffer from a slight decrease in detection accuracy. While SSD and Centernet algorithms achieve detection accuracy above 90%, their large model parameter sizes are not conducive to edge computing. As for convolutional modules such as Bifusion [49], C2f, and ConvNext, their balance between detection accuracy and model size is lower than that of YOLOv5s.

The comparison reveals that the application scenario directly influences the suitability of network models. In the non-coal foreign object detection task in coal mines, the sole introduction of attention mechanisms or network modifications can disrupt the stability of the original network, leading to varying degrees of loss in recognition accuracy and model size.

**Figure 15:**
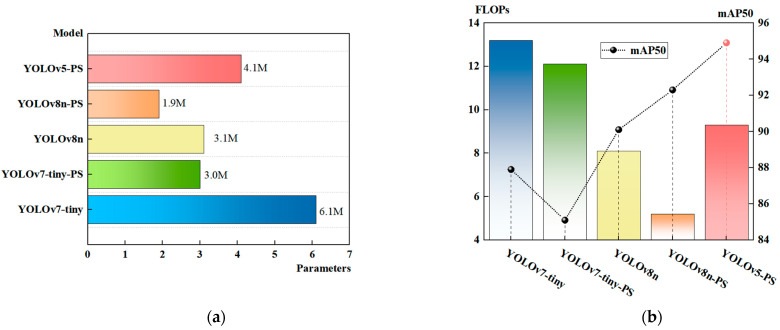
The results of the improved YOLO series of algorithms. (**a**) Parameter results; (**b**) mAP50 and FLOPs results.

In addition, YOLOv7 [50] and YOLOv8 are the two latest algorithms in the YOLO series. YOLOv7 replaces the C3 module with the ELAN module, allowing the network to learn more features and exhibit stronger robustness. The major difference between YOLOv8 compared to the fifth generation is the introduction of the C2f module, which adds more skip connections in the backbone and neck of the network. This enables YOLOv8 to obtain richer gradient flow information while maintaining its lightweight nature. We expect to achieve a similar reduction in parameters and computational complexity in the YOLOv7 and YOLOv8 models through the proposed method in this paper. Therefore, we replaced the regular convolutions in the lightweight YOLOv7-tiny and YOLOv8n models with partial convolutions. Additionally, we embedded the SimAM attention mechanism at the end of the feature extraction layers. The results are presented in Table 10 and Figure 15.

By comparing the results, it can be observed that, although the YOLOv7-tiny model has reduced the number of parameters and computational complexity (parameters: 3 M; FLOPs: 12.1 G), the detection accuracy has dropped by 2.8 percentage points. This could be attributed to the limitations of the tiny model itself and the significant differences between YOLOv7 and the fifth-generation models in terms of overall network architecture. Further optimization is required to enhance the adaptability of the proposed improvement method to YOLOv7. On the other hand, the results obtained with the YOLOv8n model were as expected, with a reduction in parameters and computational complexity by 38.7% and 27.6%, respectively. Although the YOLOv8n network model exhibits excellent lightweight performance, it currently falls short in achieving high detection accuracy. This limitation may be due to the model being in the laboratory stage and not yet deployed in underground coal mine applications, indicating the need to improve network stability and anti-interference capabilities.

### 4.4. Analysis of Ablation Experiment Results

In order to assess the reliability of each component in the improved algorithm, we conducted brief ablation experiments using the same dataset, software, hardware devices, and environmental configurations. The experimental results are presented in Table 11.

In this study, ablation experiments were conducted using YOLOv5s as the baseline. The original CIoU loss function was replaced with the proposed SIoU loss function, resulting in a 0.4% improvement in non-coal foreign object detection accuracy without any increase in parameters and computational complexity. The introduction of the SimAM attention module enhanced the recognition accuracy of non-coal foreign objects in complex mining environments, resulting in a 1.5% increase in mAP and a 6.1% reduction in model computational load. After reconstructing the C3 module with the lightweight convolutional PConv, the network reduced the demand for memory access and eliminated computational redundancy. As a result, the recognition accuracy improved by 0.7%, and the model parameters and inference time decreased by 43.1% and 56.1%, respectively. Additionally, the model computational load was reduced by 44.8%.

After incorporating various improvement modules, the recognition accuracy for non-coal foreign objects reached 94.9%. Compared to YOLOv5s, the improved model exhibited a 2.5% increase in recognition accuracy while reducing the model parameters and inference time by 43.1% and 54.1%, respectively. Additionally, the model’s computational load decreased by 43.6%. These results demonstrate the reliability of the proposed improvement algorithm, indicating that the enhanced model can efficiently and accurately detect foreign objects in conveyor belts. 

### 4.5. Analysis of Generalization Performance Results

**Figure 16:**
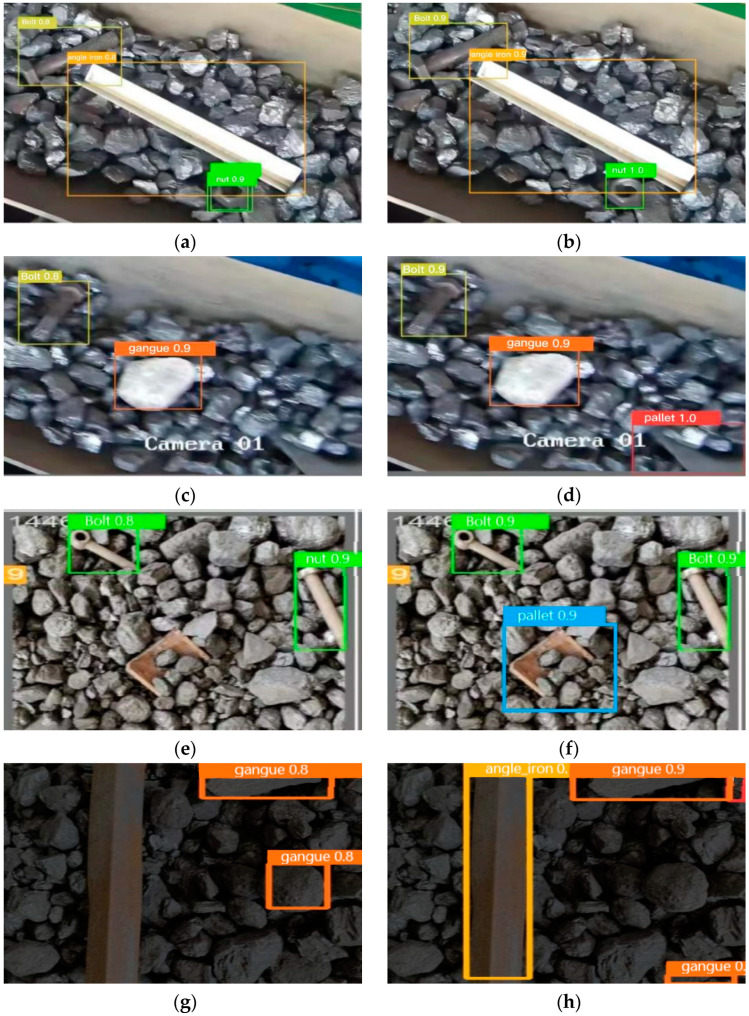
Partial recognition results. (**a**–**d**) Recognition results before and after the improvement of DataI; (**e**–**h**) recognition results before and after improvement of DataII. The left column shows the recognition results before improvement, and the right column shows the results after improvement.

Obtaining good results on a single dataset instills confidence in the model’s generalization capabilities to other non-coal foreign object datasets. However, due to the specificity of the application scenario, there is currently no publicly available dataset for foreign object detection on conveyor belts. Introducing foreign objects into a coal mine conveyor belt artificially would violate safety regulations. Taking safety factors into consideration, the validation was conducted at the National Joint Local Engineering Laboratory in Taiyuan, Shanxi Province, China. Additionally, this study further evaluated the generalization performance of the new model by using the dataset from [18]. The datasets are labeled as DataI and DataII, and the evaluation results are presented in Table 12 and Table 13. Partial recognition results are shown in Figure 16.

It can be observed that, compared to YOLOv5s, the improved model achieved an increase in recognition accuracy of 2.6% in DataI, with model parameter reduction and inference time reduction of 27.8% and 37.5%, respectively. In DataII, the recognition accuracy improved by 4.5%, while the model parameter and inference time were reduced by 41.4% and 17.3%, respectively. In addition, it can be seen from Figure 16a–d that the improved model has fewer redundant detection frames for small foreign objects and a lower rate of missed detection for corner foreign objects. From Figure 16e–h, it can be seen that the improved model demonstrates more remarkable performance in detecting buried anomalies and in low-light conditions.

### 4.6. Discussion

**Figure 17:**
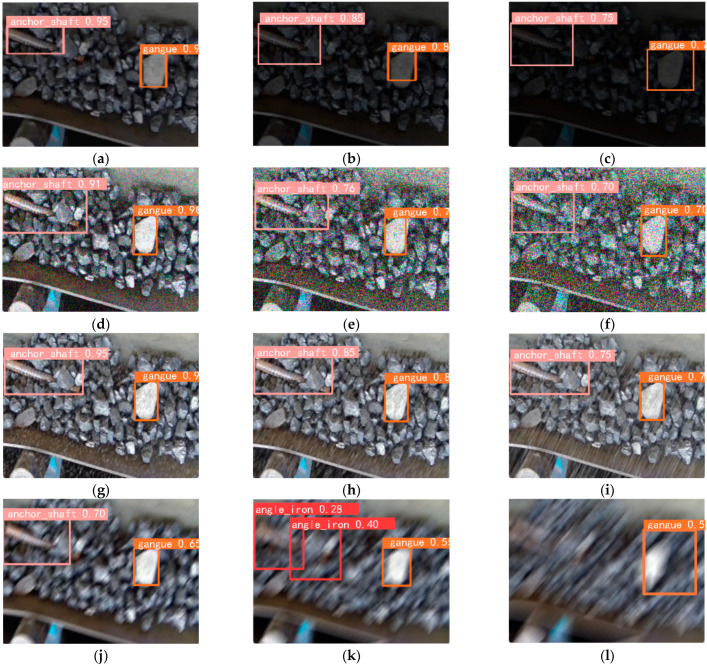
Results under different conditions. (**a**–**c**) Low light condition; (**d**–**f**) noise condition; (**g**–**i**) mist condition; (**j**–**l**) motion blur condition.

1. Currently, most lightweight methods applied in conveyor belt anomaly recognition and detection primarily focus on improving the backbone network. In contrast, this study not only improves the backbone layer but also “slims down” the neck region. In terms of test results, this study introduces lightweight measures to the backbone and neck of the original YOLOv5 network, resulting in a reduction in the parameter amount and model complexity. This approach achieves a significant improvement in detection speed while ensuring detection accuracy, leading to a prediction speed of 92 FPS for the improved YOLOv5 network. Under equivalent hardware conditions, this prediction speed surpasses YOLOv7, YOLOv8s, and other enhanced networks.
  The differences between the methods of this study and those of other researchers are shown in Table 14. Specifically, in terms of the richness of foreign object types, as compared to [14,15,18,19], the approach presented in this study exhibits superior performance in terms of both detection speed and accuracy by providing a more detailed classification of foreign objects transported (including six common types). In terms of the comparison of network model parameters and computational complexity, as compared to [16,17], the improved model in this study, although not achieving the highest accuracy, exhibits outstanding parameter efficiency (4.1 M) and FPS (92.5), which are more favorable for edge devices with limited computational capabilities. It is worth noting that the improved model in this study exhibits lower detection accuracy compared to the R-CNN network. This is partly due to the inherent limitations of the YOLO one-stage algorithm, which prioritizes faster detection speed at the expense of a certain level of accuracy. Additionally, the limitations in sample types and quantities during model training and inference require more attention to be given to complex foreign objects, which, to some extent, leads to a decrease in detection accuracy.
2. The inference speed of the same algorithm varies on different hardware. Faster reasoning through hardware with poor computing power is what we want because it will reduce costs. However, due to certain constraints in this study, the experimental training models were conducted using GPU RTX3080 processors, which slightly exceed the computational hardware commonly found in actual coal mine conditions. Therefore, further consideration is needed to evaluate the recognition performance of the network model on different processors.
3. In order to assess the impact of different underground coal mine conditions on the network’s detection results, we conducted corresponding image processing techniques, as shown in Figure 17 (the left column represents minor processing, the middle column represents moderate processing, and the right column represents severe processing).

From the perspective of the objective performance of the object detection network, thanks to data augmentation techniques and the dark channel dehazing algorithm, the object detection network exhibits strong adaptability to changes in brightness and remarkable effectiveness in recognizing noisy and hazy conditions. However, motion blur has a significant impact on the detection results. Firstly, as the blur level increases, the network fails to detect all potential objects in the image. Secondly, there is a notable deviation between the predicted values of the target’s size and position and their actual values, which may lead to missed detection and false positives. In this scenario, if the experimental results are transmitted to the corresponding sorting devices to retrieve non-coal foreign objects, it may result in equipment failure. Therefore, it is necessary to reasonably shorten the exposure time of the detection equipment to reduce the impact of motion blur. Of course, in the production process of the early dataset, it is a better solution to artificially blur the image by using image processing technology, which can also help to solve this problem.

## 5. Conclusions and Future Work

In this paper, we propose a novel method for fast and lightweight foreign object detection in belt conveyors based on YOLOv5. Through experimental validation, the following conclusions are drawn: compared to the baseline YOLOv5s, the improved model reduces the model parameters, inference time, and computational cost by 43.1%, 54.1%, and 43.6%, respectively, while achieving a maximum prediction speed of 90.2 FPS. In addition, the proposed method demonstrates excellent performance in new datasets and other object detection methods, and it is also applicable in the latest model, YOLOv8. It is hoped that the method proposed in this paper will be helpful to more developers and researchers in the recognition and detection of foreign objects on conveyor belts.

However, there are still some limitations to be acknowledged. Due to the illegality of deliberately introducing foreign objects onto working coal conveyor belts, field testing was not conducted in this study to further evaluate the algorithm’s generalization capabilities. In future work, on the one hand, we will focus on optimizing the detection results of hardware devices with low computing power, that is, conduct reasoning tests under limited computing power conditions to better reflect the superiority. In addition, in practical applications, the inevitable motion vibration caused by the motion of the inspection robot will affect the final detection accuracy. Therefore, another part of our future research will focus on mitigating the effects of vibration ambiguity and further optimizing detection methods on state-of-the-art models.

## Figures and Tables

**Table 1:** Hardware device parameters.

Device Name	Parameters
Mining inspection robot shooting frame rate	50 FPS
Shooting resolution	1920 × 1080
Belt conveyor belt speed	4 m/s

**Table 2:** Environment configuration.

Configuration Name	Configuration Parameter
Computing system	Windows11
CPU	11th Gen Intel(R) Core(TM)i7-11800H 2.30 GHz
GPU	NVIDIA GeForce RXT3080 Laptop
Python	3.7
Pytorch	1.12.0
Cuda	11.3

**Table 3:** Mean value results before and after image preprocessing.

	PSNR Value	Information Entropy	SSIM Value
Original images	21.0 ± 0.12	10.0 ± 0.25	0.70 ± 0.10
Pre-processed images	29.0 ± 0.85	16.0 ± 0.15	0.90 ± 0.08

**Table 4:** C3-P module position reliability verification experiment.

A1	A2	A3	A4	B1	B2	B3	B4	mAP (%)	Parameters (M)	Inference (s)	FLOPs (G)
								92.4	7.2	0.0089	16.5
**√**								**92.3**	**7.2**	**0.0081**	**15.8**
√	√							92.4	7.2	0.0079	14.3
√	√	√						92.4	7.1	0.0078	13.1
**√**	**√**	**√**	**√**					**92.6**	**7.0**	**0.0069**	**12.5**
√	√	√	√	√				92.5	6.9	0.0071	12.7
√	√	√	√	√	√			92.7	5.9	0.0055	11.6
√	√	√	√	√	√	√		92.9	4.7	0.0049	10.5
**√**	**√**	**√**	**√**	**√**	**√**	**√**	**√**	**93.1**	**4.1**	**0.0043**	**9.1**

**Table 5:** Comparison of C3-P and C3-D detection results.

	mAP (%)	Parameters (M)	Inference (s)	FLOPs (G)
YOLOv5s	92.4	7.2	0.0089	16.5
YOLOv5s-C3-D	**94.2**	7.0	0.0081	16.0
YOLOv5s-C3-P	93.1	**4.1**	**0.0043**	**9.1**

**Table 6:** Precision before and after improvement.

Category	P before Improvement (%)	P after Improvement (%)
Bolt	94.3	98.8
Angle iron	97.3	99.2
Anchor shaft	97.7	98.0
Coal gangue	98.0	97.8
Pallet	86.9	86.8
Nut	96.2	96.5

**Table 7:** Recall before and after improvement.

Category	R before Improvement (%)	R after Improvement (%)
Bolt	81.8	93.9
Angle iron	86.0	86.1
Anchor shaft	93.5	93.3
Coal gangue	96.9	98.4
Pallet	78.7	87.1
Nut	90.5	92.9

**Table 8:** mAP before and after improvement.

Category	mAP before Improvement (%)	mAP after Improvement (%)
Bolt	90.0	97.7
Angle iron	91.3	93.0
Anchor shaft	96.9	97.8
Coal gangue	97.7	98.5
Pallet	85.8	86.6
Nut	92.3	95.7

**Table 9:** Accuracy, parameters, and inference speed before and after improvement.

	**mAP (%)**	**Parameters (M)**	**Inference (s)**	**FLOPs (G)**
YOLOv5s	92.4	7.2	0.0089	16.5
YOLOv5m	92.9	21.2	0.0224	49.0
YOLOv5l	94.5	46.5	0.0430	109.1
YOLOv5x	**96.9**	86.7	0.766	205.7
YOLOv5s-PS	94.9	**4.1**	**0.0043**	**9.1**

**Table 10:** Improved YOLO series algorithm results (foreign object types in%, parameter quantities in M, FLOPs in G).

Model	Pallet	Anchor Shaft	Gangue	Angle Iron	Bolt	Nut	mAP50	Parameters	FLOPs
YOLOv7-tiny	83.5	92.7	92.5	91.3	93.8	91.2	**87.9**	6.1	13.2
YOLOv7-tiny-PS	80.9	93.1	92.9	92.0	91.8	90.2	85.1	**3.0**	**12.1**
YOLOv8n	71.4	93.7	92.1	93.4	95.1	92.9	90.1	3.1	8.1
YOLOv8n-PS	86.6	93.7	96.5	95.1	94.5	93.1	92.3	**1.9**	**5.2**
YOLOv5-PS	84.5	97.8	98.5	97.6	97.3	95.7	**94.9**	4.1	9.3

**Table 11:** Results of ablation experiments.

SIoU	SimAM	C3-P	mAP (%)	Parameters (%)	Inference (s)	FLOPs (G)
			92.4	7.2	0.0098	16.5
**√**			**92.8**	**7.2**	**0.0099**	**16.3**
	√		93.1	7.2	0.0098	16.0
		√	93.1	4.1	0.0043	9.1
**√**	**√**		**93.9**	**7.2**	**0.0101**	**15.5**
	√	√	94.6	4.1	0.0043	16.3
√		√	94.1	4.1	0.0045	9.2
**√**	**√**	**√**	**94.9**	**4.1**	**0.0045**	**9.3**

**Table 12:** Comparison of the effect of DataI before and after improvement.

DataI	mAP (%)	Parameters (M)	Inference Speed (s)
YOLOv5s	92.4	7.4	0.0089
YOLOv5s-PS	**94.9**	**4.7**	**0.0054**

**Table 13:** Comparison of the effect of DataII before and after improvement.

DataII	mAP (%)	Parameters (M)	Inference Speed (s)
YOLOv5s	94.6	7.0	0.0098
YOLOv5s-PS	**99.1**	**4.1**	**0.0081**

**Table 14:** Comparison with other existing methods.

Method	Foreign Object Category	mAP (%)	FPS	Parameters (M)
Improved R-CNN [15]	Coal gangue	**98.3**	-	61.0
Improved SSD [17]	Anchor rods, Wooden poles, Wooden blocks, Large coal	90.2	43.1	24.2
Improved CenterNet [20]	Gangue, Iron (channel steel, anchor rod, drill bit, and I-beam)	55.2	20	32.7
Improved YOLOv3 [16]	Wood; Bolt; Iron sheets	92.6	-	31.1
Improved YOLOv4 [18]	Gangue, Wood, Bolt, Iron sheets, Angle iron, Iron rod;	93.7	70.1	6.5
Improved YOLOv5 [19]	Anchor rod; Angle iron; Wood; Gangue; Large coal	**95.0**	56.5	7.2
Improved YOLOv7 [21]	Gangue, Bolt, Wood, Large coal, Iron	93.1	40.0	37.1
Our improved methods	Gangue; Bolt; Angle iron; Anchor shaft; nut; pallet	94.9	**92.5**	4.1

## Data Availability

The data presented in this study are available on request from the corresponding author.
